# Post-failure deformation mode switching in volcanic rock

**DOI:** 10.1098/rsos.240792

**Published:** 2024-08-28

**Authors:** Jamie I. Farquharson, Michael J. Heap, Lucille Carbillet, Patrick Baud

**Affiliations:** ^1^ Institute for Research Administration, Niigata University, Ikarashi 2-8050, Nishi-ku, Niigata 950-2181, Japan; ^2^ Research Institute for Natural Hazards and Disaster Recovery, Niigata University, Ikarashi 2-8050, Nishi-ku, Niigata 950-2181, Japan; ^3^ Université de Strasbourg, CNRS, Institut Terre et Environnement de Strasbourg, UMR 7063, 5 rue Descartes, Strasbourg F-67084, France; ^4^ Institut Universitaire de France (IUF), Paris, France; ^5^ Laboratory of Experimental Rock Mechanics, Ecole Polytechnique Fédérale de Lausanne, Lausanne, Switzerland

**Keywords:** compaction, dilatancy, brittle-ductile transition, andesite, rock deformation, permeability

## Abstract

Beyond a threshold applied compressive stress, porous rocks typically undergo either dilatant or compactant inelastic deformation and the response of their physical properties to deformation mode is key to mass transport, heat transport and pressure evolution in crustal systems. Transitions in failure modes—involving switches between dilatancy and compaction—have also been observed, but to date have received little attention. Here, we perform a series of targeted mechanical deformation experiments on porous andesites, designed to elucidate complex post-failure deformation behaviour. By investigating a sample suite and effective pressure range that straddles the transition between positive and negative volumetric responses to compression, we show two post-failure critical stress states: a transition from compaction to dilation ( $C^{*}\prime$ ), and a transition from dilation to compaction, which we term $C\prime^{*}$ . We demonstrate that multiple switches in deformation mode can be driven by stress application under conditions relevant to the shallow crust. While the effect on fluid flow properties of compaction-to-dilation switching may be masked by a net reduction in sample porosity, samples that underwent dilatant-to-compactant failure mode switching exhibited an increase in permeability of approximately two orders of magnitude, despite only slight net volumetric change. Such a substantial permeability enhancement underscores the importance of post-failure deformation in influencing solute and heat transfer in the crust, and the generation of supra-hydrostatic fluid pressures in volcanic environments.

## 1. Introduction

Permeability is a critical parameter in the crust because it is a process-limiting property [1,2]. For example, permeability determines the capacity of a geologic system for mass and heat transfer [3], and governs the generation of supra-hydrostatic fluid pressures [4], which is of fundamental importance to volcanic activity and associated hazards [5–7]. Permeability can vary by multiple orders of magnitude in volcanic systems, in particular as a function of porosity [8–13]. Porosity and the connectivity of porosity can evolve in both space and time; models and experiments demonstrate that this can lead to a corresponding change in permeability [14–19].

The mechanical response of subsolidus volcanic rock to applied differential stress may be dilatant (porosity-increasing, characterized by formation of localized fracturing) or compactant (porosity-decreasing, characterized by cataclasis and pore collapse). Whether or not a rock is dilatant or compactant depends on several parameters, including the pre-existing pore structure of the rock and the chemical, thermal and mechanical conditions under which it is deformed [20,21]. Generally, at low confining pressures—which we may broadly equate to shallow crustal depths—and low initial porosity $\phi_{0}$ , we may expect shear localization to occur in volcanic rock, whereas ductile (compactant) behaviour is facilitated by relatively higher confining pressures (i.e. deeper environments) and higher $\phi_{0}$ [17,22–26]. Whether parts of a volcanic edifice—subject to a broad range of local and regional stresses—undergo dilation or compaction as a result of *in situ* stresses is critical in terms of the structural stability of a volcano [27,28] and its capacity for outgassing volatiles [6], in turn linked to overpressure generation and attendant volcanic hazards. Drilling data reveal that the internal structure of the upper conduit architecture of silicic to intermediate volcanoes is often characterized by intense fracturing and sheared margins in the upper few kilometres, noted for example at Unzendake, Japan [29] and Nigorikawa Caldera, Japan [30]. Such a diversity of textures may well be indicative of multiple overprinting of brittle and ductile deformation mechanisms, but such processes have received little attention in laboratory-based studies.

The influence of deformation—particularly post-failure deformation—on the fluid transport properties of volcanic materials is not well studied. Fortin *et al*. [31] showed experimentally that the permeability of a low-porosity basalt was enhanced following macroscopic shear fracture, but also revealed that the direction and magnitude of permeability evolution was not constant during deformation. Farquharson *et al*. [32] performed triaxial deformation experiments on suites of variably porous volcanic rocks, demonstrating progressive porosity and permeability increase relative to the initial conditions following post-failure strain accumulation in the brittle regime. On the other hand, Heap *et al*. [16] report a net decrease in permeability for samples of trachyandesite undergoing continued deformation following brittle failure. In the ductile regime, post-failure strain accumulation in volcanic rocks has been associated with a decrease in porosity and permeability in andesite [16,17,33]. Alam *et al*. [34] investigated permeability evolution of Shikotsu welded tuff, finding that permeability decreased monotonically with triaxial compression (both in dilation and compaction) and that the rate of permeability decrease was tied to the effective pressure under which deformation was performed. More complex behaviour was observed by Farquharson *et al*. [35]: at axial strain ( ϵ ) less than 0.05, andesite samples exhibited an increase in permeability by approximately an order of magnitude relative to their initial state, despite a decrease in porosity; beyond ϵ = 0.05, both porosity and permeability tended to decrease with increased strain accumulation.

Previous studies that have investigated the post-failure evolution of permeability of volcanic materials have primarily done so under conditions whereat the post-failure porosity evolution has been monotonic (i.e. brittle failure followed by progressively dilatant deformation, or progressive inelastic compaction during ductile deformation). Typically, brittle triaxial experiments are arrested shortly after the initial stress drop associated with macroscopic fracturing, while ductile experiments are stopped after a few per cent of sample shortening (strain accumulation). However, under certain triaxial conditions and sufficient inelastic strain accumulation, it has been observed that deformation can result in non-monotonic porosity evolution, manifest as a switch from compaction to dilation. This has been observed frequently in limestones [20,36], but also sandstones [20,37] and andesite [17,35]. Such transitional behaviours remain understudied in volcanic rocks, despite the fact that the conditions under which they are achieved are relevant to shallow volcanic environments. To address this knowledge gap, we perform a series of targeted mechanical deformation experiments on volcanic rocks of intermediate porosity (approx. 0.14−0.21), designed to showcase post-failure transitional behaviour and quantify the corresponding evolution of fluid transport properties.

## 2. Material and methods

The primary material used throughout this study is an andesitic lava collected from Mount Ruapehu in the Taupō Volcanic Zone (Aotearoa New Zealand). A hand sample was collected on the northern flank of the volcano [38], part of the Whakapapa Formation—the youngest unit of the Ruapehu edifice [39]. The site is culturally and ecologically protected: sampling was confined to a small block, leaving no trace of sampling, in line with sample permit guidelines and cultural consideration. From the initial block, a suite (13) of 20 mm diameter cores were prepared and precision-ground to a length of 40 ± 1 mm so that their end faces were flat and parallel. After drying the samples under vacuum, porosity was measured by helium pycnometry, and gas permeability was measured using the set-up described in Farquharson *et al*. [40]. This andesite (block R10) was selected primarily due to its intermediate porosity (mean $\phi_{0}$ ~ 0.16), which falls between the end-member sample sets used previously to investigate exclusively dilatant [41] or exclusively compactant [35] post-failure processes. More importantly, the sample porosities in this study fall within the range of modal porosities of compiled field data [10,42,43], suggesting that the sample behaviours are relevant to a significant proportion of the edifice, by volume. An additional single experiment was performed on a sample of La Lumbre andesite (LLB) from Volcán de Colima (Mexico), from the same suite as described in Farquharson *et al*. [35]. This sample was also characterized as described above. Ruapehu and Volcán de Colima have many characteristics in common with each other (and more broadly with many convergent margin volcanoes), including their overall geomorphology and their histories of collapse events, cyclic eruptive behaviour and periods of dome effusion. Moreover, recent eruptive products have been compositionally similar: the dome-forming lavas from Volcán de Colima (58−61 wt% SiO_2_ [44]) fall within the compositional range of the Whakapapa Formation unit (57−66 wt% [39]). The lavas from both systems tend to be microstructurally complex [38,44]: highly crystalline, porous and pervasively microcracked.

Core samples were soldered into copper foil and saturated under vacuum with deionized water, then deformed in the triaxial rig at the Strasbourg Institute of Earth and Environment in France (a schematic of the device and description of the method are given in Farquharson *et al*. [35]). During deformation, we assume an effective pressure ⟨p⟩ [45,46] such that $\left\langle p\right\rangle =\sigma_{3}-\alpha p$ , where $\sigma_{3}$ is the confining pressure ( $p_{c}$ ), p is the interstitial pore fluid pressure and α is the Biot–Willis coefficient [47,48]. By performing triaxial deformation experiments with nominally the same ⟨p⟩ but different p , Farquharson *et al*. [49] show that for a Volcán de Colima andesite, α can be assumed to equal 1. Given the similarities described above, we further assume that this is the case for all andesites in this study. Values of effective pressure $\left\langle p\right\rangle$ of 10, 30, 50 or 70 MPa were imposed on the samples (with a pore pressure p of 10 MPa). To deform samples, a differential stress ( $\sigma_{1}-\sigma_{3}$ ) was applied in the direction of the sample axis by advancing a servo-controlled axial piston at a constant strain rate of 10^−5^ s^−1^, sufficiently slow to ensure that the sample remains fluid-saturated and drained throughout the experiment [50]. Confining and pore pressures are also servo-controlled; accordingly, variations in sample pore volume during deformation are recorded by the pore fluid actuator, as it compensates for pore fluid pressure changes [51]. Normalized to the sample volume, this response corresponds to porosity change δϕ , which for porous rock can be considered approximately equivalent to the volumetric strain (see [52]). Deformation was allowed to continue post-failure to a target axial strain. The target value varied between experiments, from ϵ= 0.02 up to a maximum of ϵ=0.25 (a 25% shortening of the sample). Because the axial piston has a finite stroke length, large amounts of strain were achieved by periodically isolating the sample within the pressure vessel and recharging the loading ram before continued deformation. In practice, this involves closing the valve that connects the pressure vessel to the axial piston system (i.e. valve 3 in the schematic of [35]) and retracting the piston towards its initial position. During this time, the piston head is not under servo-control, and thus some drift is anticipated and corrected for manually: this can manifest as transient minor stress reductions in the raw data, which can be accounted for during data processing. After deformation, samples were unloaded and their permeability remeasured (see [35]). In the following, we adopt the convention that compressive stress and compactant strain are positive. All experiments were carried out at room temperature. Select deformed and undeformed samples were imaged using a scanning electron microscope (SEM). SEM analysis of the Ruapehu andesite reveals a porphyritic juvenile andesite with abundant tabular plagioclase phenocrysts set in a dense glassy microlitic matrix. The main pore shape families are (i) approximately circular, with characteristic length-scales of the order of 100 μm, and (ii) highly amœboid and frequently elongate pores with diameter of the order of 1000 μm. However, these end-members fall on a spectrum and there is no clear threshold between the two. Pores are variably interconnected by microcracks.

## 3. Results and discussion

### 3.1. Influence of effective pressure and porosity on failure and deformation

**Figure 1.**
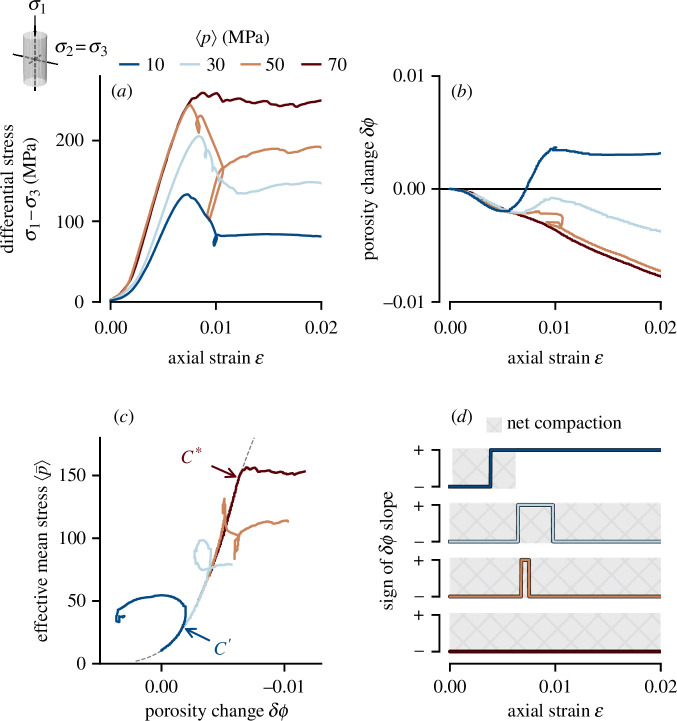
(*a*) Differential stress versus axial strain for andesite samples (R10–7, –15, –8 and −16) deformed at $\left\langle p\right\rangle =$ 10, 30, 50 and 70 MPa, respectively (indicated by colour). (*b*) The same samples as (*a*), showing porosity change as a function of axial strain. (*c*) Effective mean stress as a function of porosity change. The data have been offset from the *y*-axis to align with the assumed hydrostatic signal (dashed line). (*d*) The sign (positive or negative) of the porosity change slope, as shown in (*b*). Shaded area indicates periods of net compaction.

Figure 1 shows representative deformation results from samples deformed under different effective pressures $\left\langle p\right\rangle$ . Figure 1*a* plots differential stress ( $\sigma_{1}-\sigma_{3}$ ) as a function of axial strain. For samples deformed at ⟨p⟩ = 10, 30 and 50 MPa, deformation is characterized by a brief concave-up signal (signifying elastic crack closure), followed by linearly increasing $(\sigma_{1}-\sigma_{3})/\epsilon$ . After rolling over to a peak value in $\sigma_{1}-\sigma_{3}$ , we observe a stress drop (from approx. 40 to over 100 MPa) followed by quasi-constant stress with additional strain accumulation. The peak stress and stress drop are associated with the generation of a shear band: visual inspection of the failed samples reveals localized failure running subparallel to the sample axis (10 MPa) or inclined to the axis (30 and 50 MPa). It is probable that post-failure, strain is accommodated by sliding on the resultant macroscopic fracture. Under effective pressure of 70 MPa, the initial part of the curve follows the same trend. However, there is no marked drop in stress, and the sample undergoes compactant (ductile) yield rather than brittle failure. In this case, no macroscopic fracture was observed in the failed sample. We note that previous studies [17,22,23,35] have highlighted compaction localization features in porous volcanic rocks: bands or zones of collapsed pores tending sub-perpendicular to the maximum principle stress $\sigma_{1}$ . Such localization is typically accompanied by transient, post-yield stress drops of a few MPa; in this study, we observe such stress drops for some but not all experiments in the ductile field, suggesting that compaction is manifest both in a distributed manner (diffuse microcracking and distributed pore collapse) and occasionally in a localized manner (a contiguous plane of collapsed pores).

In figure 1*b*, we show porosity change δϕ as a function of strain ϵ for the same experiments shown in figure 1*a*. For effective pressure $\left\langle p\right\rangle =10$ MPa, we observe an initial decrease in porosity (compaction associated with elastic crack closure), a (pre-failure) switch from compaction to dilation whereat new cracking exceeds the rate of compaction, followed by a relatively constant value of porosity change with strain. The volumetric deformation curves (figure 1*b*) confirm that the deformation behaviour of samples deformed at ⟨p⟩>10 MPa is net compactant: the deformed samples are always less porous than at the start of the experiment. However, whereas at ⟨p⟩ = 70 MPa (the ductile experiment), δϕ decreases monotonically with strain ϵ , this is not the case for the intermediate effective pressures (30 and 50 MPa). This is visualized in figure 1*c*, where we show the effective mean stress $\left\langle \overset{-}{p}\right\rangle$ (a function of all stresses acting on the sample: $\left[\sigma_{1}+2\sigma_{3}\right]/3-p$ ) against δϕ . In cases where the signal deviates leftwards from this curve—a stress threshold called *C′,* which represents the onset of dilatant microcracking—we can define eventual failure in the brittle regime (demarcated by the stress, strain coordinates corresponding to the maximum differential stress $\sigma_{p}$ , figure 1*a*). The corresponding macroscopic ‘failure’ in the ductile field is represented by a critical stress point termed $C^{*}$ , which is the onset of shear-enhanced compaction. Graphically, this is the point at which the stress–strain curve deviates to the right of the hydrostatic signal (e.g. figure 1*c*). The volumetric response of the samples during deformation is further visualized in figure 1*d*, where we show the sign of the porosity change slope (figure 1*b*): decreasing or increasing porosity is denoted by – or + , respectively, and the shading indicates whether the sample is net compactant (i.e. whether it has a lower porosity than at the start of the experiment $\phi_{0}$ at any given point during deformation).

At ⟨p⟩ = 10 MPa, the sample is entirely dilatant. The failure of the sample, described by $\sigma_{p}$ , is a function of growth and coalescence of microcracks into a failure plane (a macroscopic fracture). At ⟨p⟩ = 70 MPa, the sample is entirely compactant and porosity continues to decrease as the sample is deformed beyond $C^{*}$ (figure 1*c,d*). At ⟨p⟩ = 30 and ⟨p⟩ = 50 MPa, both samples exhibit an initial dilatant period (deviations to the left on figure 1*c*) leading to a peak stress: failure in the brittle regime. However, unlike the experiment performed at ⟨p⟩ = 10 MPa, which continued to dilate, continued post-failure strain accumulation at ⟨p⟩ = 30 and ⟨p⟩ = 50 MPa results in a transition from dilatant to compactant behaviour. As evident in figure 1*b–d*, these samples are net compactant despite having failed in the brittle field, and are characterized by *C*′ rather than $C^{*}$ . This transitional behaviour will be discussed further in §3.3. Deformation conditions and results are given in table 1.

**Table 1.** Deformation conditions and results.

sample	effective pressure (MPa)	pore pressure (MPa)	peak stress σ*_p_* (MPa)	C* (MPa)	C*′	*C*′*
R10–3	10	10	145.99		╳	╳
R10–4	50	10	261.8		╳	◯
R10–5	50	10	279.53		╳	✓
R10–6	50	10	210.22		╳	◯
R10–7	10	10	143.07		╳	╳
R10–9	50	10	267.06		╳	✓
R10–10	10	10	142.13		╳	╳
R10–11	10	10	133.3		╳	╳
R10–12	50	10	243.9		✓	◯
R10–13	10	10	130.8		✓	✓
R10–15	30	10	205.39		╳	✓
R10–16	70	10		214.3	╳	╳
R10–17	70	10		254.94	╳	╳
LLB-2	30	10		54.87	✓	╳

✓critical stress state observed; ╳ critical stress state not observed; ◯ intermediate stress behaviour.

**Figure 2.**
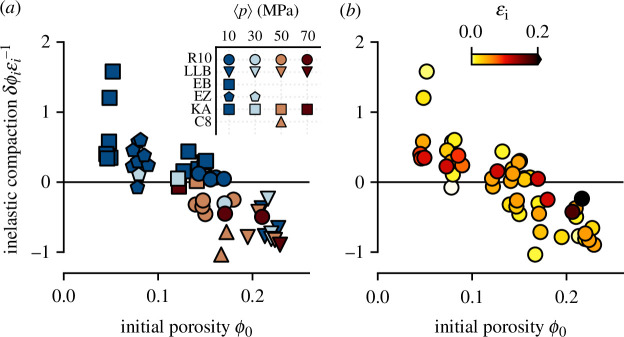
(*a*) Inelastic compaction factor as a function of initial porosity for samples of this study (R10) and compiled volcanic data from [32] (EB, Etna basalt, EZ, El Zarco andesite, KA, Kumamoto andesite) [17]; (C8, San Antonio andesite); and [35] (LLB, La Lumbre andesite). Colours represent ⟨p⟩ , as in figure 1. (*b*) As (*a*), but differentiating data by inelastic strain $\epsilon_{i}$ .

From the inelastic components of axial strain $\epsilon_{i}$ and porosity change $\delta \phi_{i}$ , we can determine an inelastic compaction factor $\delta \phi_{i}\epsilon_{i}^{-1}$ [53,54]. This parameter quantifies the amount of change of sample porosity for additional increments of inelastic strain. In figure 2*a*, we plot this parameter—calculated from these experiments as well as compiled data on volcanic rocks from [17,32,35]—against the initial sample porosity $\phi_{0}$ . Some of the volcanic systems represented in the compilation are good analogues for each other (notably Ruapehu and Colima). They also comprise particularly strong analogues for many other active andesitic stratovolcanoes—including Mount Rainier (USA), Ulawun (Papua New Guinea), Llaima (Chile), Ijen (Indonesia) and Galeras (Colombia)—when their eruptive histories are considered alongside geological and geochemical characteristics [55,56]. More importantly in the context of this study, however, is the fact that these samples have been deformed using the same experimental protocol, on the same apparatus and have received the same data treatment. While variability exists within each sample suite due in part to the different degrees of axial strain (figure 2*b*) and effective pressures associated with each test [57], the influence of initial porosity $\phi_{0}$ is clear: at low starting porosities (< 0.1), samples tend to exhibit $\delta \phi_{i}\epsilon_{i}^{-1}>0$ whereas at higher starting porosities (> 0.15), we observe $\delta \phi_{i}\epsilon_{i}^{-1}<0$ . This is as expected: porosity is a key parameter influencing whether a rock will compact or dilate in response to applied stress [20]. Similarly, figure 2*b* shows that a tendency towards zero exists with increasing inelastic strain accumulation, irrespective of whether the inelastic compaction factor is initially positive or negative. A key feature of our experimental design is that our new data encompass the transition between $\delta \phi_{i}\epsilon_{i}^{-1}$ > 0 and $\delta \phi_{i}\epsilon_{i}^{-1}$ < 0. For R10 andesite (circles in figure 2*a*) samples deformed at ⟨p⟩ = 10 MPa, we observe $\delta \phi_{i}\epsilon_{i}^{-1}>0$ , meaning that for additional increments of inelastic axial strain, sample porosity increases. For R10 samples deformed at $\left\langle p\right\rangle \geq$ 30 MPa, the inelastic compaction factor $\delta \phi_{i}\epsilon_{i}^{-1}<0$ (figure 2*a*), meaning that for additional increments of inelastic axial strain, sample porosity decreases.

### 3.2. Post-failure transition from compaction to dilation

Porosity is a first-order parameter dictating deformation behaviour of porous rock [20], including porous volcanic rock [21]. However, as demonstrated in figure 1*b–d*, porosity is subject to change with continued strain. Theoretically, after a given amount of compaction, porosity may be reduced to a point whereat any additional deformation of a compacting material is accommodated by macroscopic fracturing (as it would be for an initially low-porosity material). The strain-dependent transition from compactant to dilatant behaviour is termed $C^{*}\prime$ , and defines the maximum compaction that a given rock can withstand. This transition has been demonstrated experimentally in sedimentary [36,37,53,58,59] and volcanic [17,35] rocks. Heap *et al*. [17] observed $C^{*}\prime$ in a porous andesite deformed at $\left\langle p\right\rangle =$ 50 MPa after an axial strain of 0.13, and a corresponding reduction in porosity δϕ of approximately 0.04. Farquharson *et al*. [35] report $C^{*}\prime$ in a porous andesite deformed at $\left\langle p\right\rangle =$ 30 MPa after an axial strain of 0.16, and a reduction in porosity δϕ of approximately 0.05.

**Figure 3.**
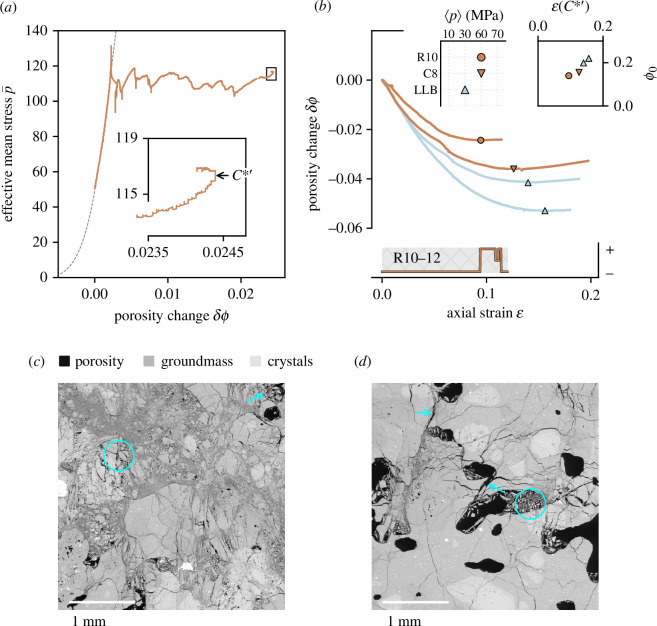
(*a*) Effective mean stress versus porosity change for andesite sample R10−12. Dashed line is as in figure 1*c*. Inset shows detail of critical stress state $C^{*}\prime$ . (*b*) Compiled porosity change curves for andesites that have reached $C^{*}\prime$ . R10−12: $\phi_{0}=0.18$ , $\left\langle p\right\rangle =50$ MPa; C8−8: $\phi_{0}=0.16$ , $\left\langle p\right\rangle =50$ MPa; LLB−13: $\phi_{0}=0.22$ , $\left\langle p\right\rangle =30$ MPa; LLB−2: $\phi_{0}=0.20$ , $\left\langle p\right\rangle =30$ MPa. Below is the sign of the porosity change slope for sample R10−12. Inset shows strain at $C^{*}\prime$ as a function of initial porosity. (*c*) SEM image of heavily comminuted groundmass (e.g. circle) and pore collapse (e.g. arrow) in R10−12, following $C^{*}\prime$ . SEM images are oriented such that the maximum principal stress during deformation is oriented vertically. (*d*) Example of interconnected porosity in R10−12, whereby pores are joined by variably coalesced microcracks (highlighted by arrows). Intra-pore spalling grains, evidence of cataclastic pore collapse, are evident towards the centre of the image (cyan circle).

In figure 3*a*, we show porosity change as a function of effective mean stress for Ruapehu andesite R10−12. Initially, porosity decreases (the porosity change signal δϕ moves rightward), equivalent to other compactant experiments presented here (e.g. figure 1*c*). However, after a reduction in sample porosity from 0.18 to 0.155 (porosity change of approximately 0.024), we observe dilatant deformation (the signal moves leftwards). This transition point, $C^{*}\prime$ , is shown in more detail in figure 3*a* (inset) and corresponds to the point where the first derivative of the volumetric deformation signal (as a function of axial strain) switches sign from negative to positive.

In figure 3*b*, we compile prior data (andesites from Volcán de Colima: C8-8 and LLB-13) with data from two new experiments (Ruapehu andesite R10−12 and Colima andesite LLB-2), plotted as porosity reduction as a function of axial strain. The transition point from compaction to dilation is marked by a symbol for each sample. To account for experimental artefacts associated with recharging the axial loading ram, we first apply a one-dimensional Gaussian filter to the deformation signal f(δϕ) , then obtain the change-point at the global minimum (i.e. f′(δϕ)=0 ). Generally, there is a correlation between the volume of porosity reduction required to achieve $C^{*}\prime$ and the associated axial strain (i.e. high porosity change requires high strain (figure 3*b*). The strain at $C^{*}\prime$ , labelled $\epsilon (C^{*}\prime )$ , is shown as a function of initial porosity $\phi_{0}$ in figure 3*b* (inset). Our data reveal a clear dependence on the initial porosity: the axial strain required for $C^{*}\prime$ increases as $\phi_{0}$ . Although the data are too few to draw additional conclusions, there also appears to be a measurable influence of effective pressure, i.e. a greater degree of strain is required to achieve the critical stress transition at lower effective pressure for samples of similar starting porosity (figure 3*b* and inset). These observations agree with results from sedimentary rocks [36,53], despite differences in the dominant micromechanical processes. In the broader context of a volcanic system, this would suggest that compactant-to-dilatant transitional behaviour may be more prevalent in the shallow volcanic edifice than previously discussed [17] ( $\left\langle p\right\rangle =30$ MPa is approximately equivalent to 1.3 km depth beneath the summit). In §4, we further discuss the effect of this transition on fluid flow properties, with attendant implications for volcanic behaviour.

Microstructurally, we observe characteristic compactant features in Ruapehu andesite sample R10−12 (mechanical data shown in figure 2*a*). These include heavily comminuted groundmass, micrometre-scale crystal pulverization (examples circled on figure 3*c*,*d*) and other evidence of cataclastic pore collapse (arrow on figure 3*c*). These are frequently overprinted or connected in places by fractures (e.g. see arrows in figure 3*d*), as described in earlier studies on volcanic rocks [17,35].

### 3.3. Post-failure transition from dilation to compaction

**Figure 4.**
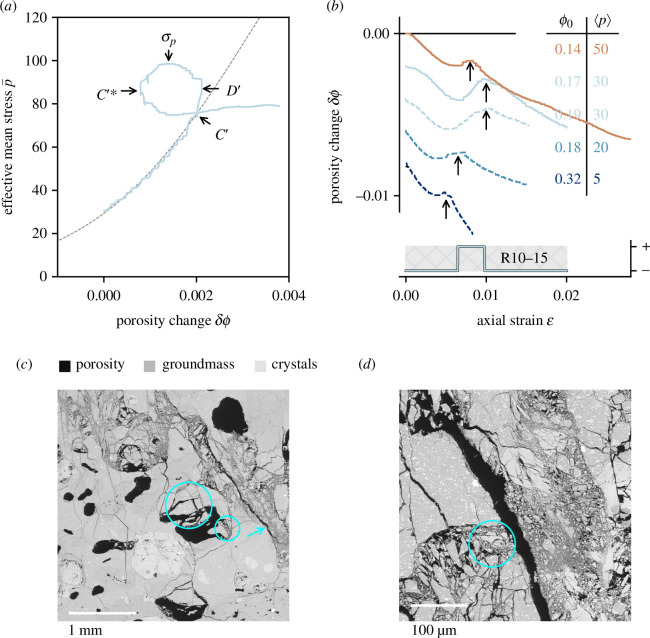
(*a*) Effective mean stress versus porosity change for sample R10−15, highlighting sequence of critical stress states including $C\prime^{*}$ . (*b*) Compiled porosity change data for R10 samples and samples of Mount St Helens dacite (dashed lines) from [61]. Note that curves are vertically offset for clarity. $C\prime^{*}$ is highlighted by an arrow in each case. Initial porosity $\phi_{o}$ and effective pressure ⟨p⟩ (in MPa) are indicated for each sample. Below is the sign of the porosity change slope for sample R10−15. (*c*) SEM image of sample R10−15, showing coalesced fractures (e.g. arrow) and evolved fault zone, surrounded by partially collapsed pores (circled). (*d*) Further detail of the fracture zone.

While the compactant-to-dilatant critical stress threshold has been observed and described in prior studies (see references in §3.2), the inverse phenomenon—a transition from dilatancy to a dominantly compactive mechanism—has not been explicitly described, to the authors’ knowledge. However, theoretically there should exist a stress state whereby a dilating sample undergoes an increase in porosity sufficient to encourage a compactive response to additional deformation, as posited by Meyer and Violay [60]. Herein, experiments were performed with the intention of demonstrating this transition: an example is shown in figure 4*a*. The porosity reduction curve as a function of effective mean stress is given for Ruapehu andesite R10−15 ( $\phi_{0}=0.14$ ; $\left\langle p\right\rangle =50$ MPa), which exhibits complex mechanical responses during prolonged deformation. The stress path is characterized by a series of critical stress states: (i) the onset of dilatancy, demarcated by $C^{\prime }$ [54]; (ii) the point at which dilation overtakes compaction, termed $D^{\prime }$ [62]; (iii) a peak stress $\sigma_{p}$ , signifiying macroscopic failure in the brittle regime; and (iv) a post-failure transition from dilation to compaction. This latter transition is $C\prime^{*}$ , and proves to be repeatable for the andesites of this study, despite inherent sample variability (figure 2*b*): this critical stress state was achieved at $\left\langle p\right\rangle =$ 10, 30 and 50 MPa (table 1). Samples that have undergone $C\prime^{*}$ exhibit shear fractures oriented obliquely to the maximum principal stress (see arrow in figure 4*c*). Fractures appear to have coalesced stepwise as dictated by pre-existing, irregularly shaped pores. The fractured zones are variably 100−1000 μm in width, with gouge formation [63] characterized by microfracturing and comminution, and evidence of translation and rotation of fragments (figure 4*c*). As highlighted in figure 4*d*, there is also evidence of cataclastic pore collapse (e.g. as highlighted by the cyan circles), typically inside or within a few hundred micrometres of the primary fracture zone. This is manifest as partially collapsed pores, with more intense cataclasis oriented towards the fracture plane (circled) (figure 4*d*). In light of the associated mechanical data, we interpret these microtextures as representing an initial fracture event followed by fracture zone evolution and thickening, before the onset of localized pore collapse driven by local porosity (and stress) change. Many of the textures observed in samples that have undergone $C^{*}\prime$ or $C\prime^{*}$ are similar (cf. figures 3*d* and 4*c*), although extensive fault gouge formation may be distinguishable from *in situ* cataclasis by observation of grain translation along the fault plane. We note that the primary observable difference is the macroscopic orientation of the failure localization plane(s): compaction localization (in the case of $C^{*}\prime$ ) is approximately perpendicular to the sample axis (figure 3*c*), whereas failure localization in the case of $C\prime^{*}$ is subparallel to the sample axis (figure 4*c*).

We emphasize that although this critical stress transition has not—to our knowledge—been formally described by previous authors, there exists evidence of volcanic samples achieving $C\prime^{*}$ in some published experimental studies. The porosity change data from [61] (their figures 4*c* and 5*c*) show this phenomenon in Mount St Helens dacite; the transition is also visible in experimental data from Volvic trachyandesite ([16], their figure 3; [21], their figure 17*b*). Moreover, figure 5*a,f* of Carbillet *et al*. [64] seemingly documents this phenomenon in synthetic samples (sintered monodisperse glass beads, analogous to monomineralic well-sorted sandstones). Data from Heap *et al*. [61] (dashed lines) are plotted alongside data from the current study (solid lines) in figure 4*b*; the critical stress state $C\prime^{*}$ is highlighted by the arrows. In comparable manner to the stress and porosity dependence of the ‘critical state line’ [17], we would anticipate the range of stresses at which the $C\prime^{*}$ phenomenon becomes evident to scale inversely with initial sample porosity, with the implication that at high initial porosities, a post-failure switch from dilatant to compactant behaviour would occur at low effective pressure, whereas at relatively lower initial porosities, a higher effective pressure would be required to instigate this post-failure transition. In sedimentary materials, evidence for a critical state $C\prime^{*}$ (as we define it) appears to be rare. More often, the brittle-ductile transition in sedimentary rocks is characterized by volumetrically neutral behaviour. In a graph of effective mean stress versus porosity change, this response is manifest as the curve following the hydrostat before stalling around a single point: continued deformation does not affect the bulk sample porosity (i.e. inelastic compaction factor $\delta \phi_{i}\epsilon_{i}^{-1}=0$ ) [20,54,57,65,66]. The corresponding stress–strain responses in those sedimentary examples reveal that the samples do not exhibit the characteristic yield point ( $C^{*}$ ) but continue to accumulate strain through cataclastic flow and/or the generation of compaction bands while accommodating a near-constant axial stress level. Tentatively, we attribute the differing behaviours between the volcanic rocks studied here and sedimentary rocks of prior studies to a difference in the characteristic flaw length-scale (e.g. pore radius of the lavas versus grainsize of the granular sedimentary samples), of the respective materials, but this remains a detail for future investigation. Given that volcanic deposits can be fundamentally granular (e.g. welded fall deposits), and that many volcanic systems overlie a sedimentary basement, we emphasize the importance of investigating post-failure deformation for a broad range of crustal materials and analogues.

**Figure 5.**
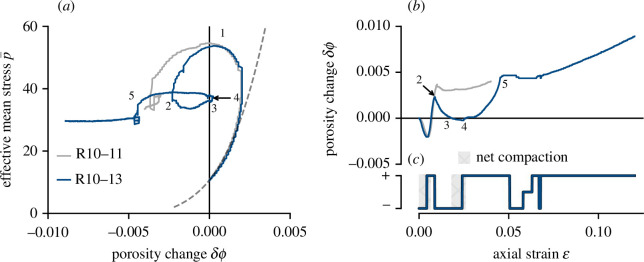
(*a*) Porosity change versus effective mean stress for andesite sample R10−13 (blue) and R10−11 (grey). Stress states 1−4 are described in the main text. (*b*) Porosity change curve for R10−13 and R10−11. (*c*) Sign of the porosity change curve for sample R10−13.

### 3.4. Multiple post-failure switches in deformation mode

Continued post-failure compaction can eventually initiate dilatant deformation (§3.2, figure 3*a–b*). Likewise, we demonstrate that post-failure dilation can precede net compaction (§3.3, figure 4*a–b*). It stands to reason that there exists a range of initial sample porosities and deformation conditions such that the inelastic compaction factor $\delta \phi_{i}\epsilon_{i}^{-1}$ (figure 2*a,b*) is equal or sufficiently close to zero that small increments of strain—characterized by a slight *increase* or *decrease* in porosity—can flip the sign of $\delta \phi_{i}\epsilon_{i}^{-1}$ , thereby prompting a slight *decrease* or *increase* in porosity sufficient to re-flip the sign of $\delta \phi_{i}\epsilon_{i}^{-1}$ , and so on. In a final deformation experiment, we seek to capture this behaviour, selecting a sample of Ruapehu andesite (R10−13) with an initial porosity of $\phi_{0}=0.17$ and deforming it at an effective pressure $\left\langle p\right\rangle =$ 10 MPa. The results are shown in figure 5, with the deformation signal of a slightly lower porosity sample (R10−11, $\phi_{0}=0.16;\left\langle p\right\rangle =10$ MPa) shown for reference.

The two samples follow a comparable stress evolution path until immediately prior to macroscopic failure in the brittle regime, with peak stresses (1 in figure 5*a*) of 131 MPa (sample R10−13) and 133 MPa (sample R10−11). Post-failure, both samples exhibit a phase of dilatant deformation, a function of continued fracture evolution and fault sliding [32]. After accumulating 0.009 axial strain ( δϕ ∼0.0024 ), sample R10−13 reaches *C*′* (transition from dilatant to compactant deformation; 2 in figure 5*a,b*), as described in §3.3, whereas sample R10−11—the grey curve—does not. For sample R10−13, compaction with continued strain accumulation is manifest in figure 5*a,b* by evolution from 2 → 3. Critically, at this point, $\phi_{0}+\delta \phi =\phi_{0}$ : the sample has exactly the same porosity as at the beginning of the experiment (again, this is not true for sample R10−11). Briefly, the sign of porosity change flips as the sample undergoes *net* compaction (3 → 4), before again crossing the δϕ=0 line and undergoing dilation. We interpret this phase (4 → 5) as reactivation of the initial macroscopic failure plane followed by fault sliding. Note that point 4 fits the definition of C*′. With continued deformation, the sample undergoes a sequence of transitions from dilation–compaction–dilation–compaction–dilation, although the net porosity change of the sample remains positive. Figure 5*c* illustrates the full sequence as a function of the sign of the slope of the δϕ signal.

**Figure 6.**
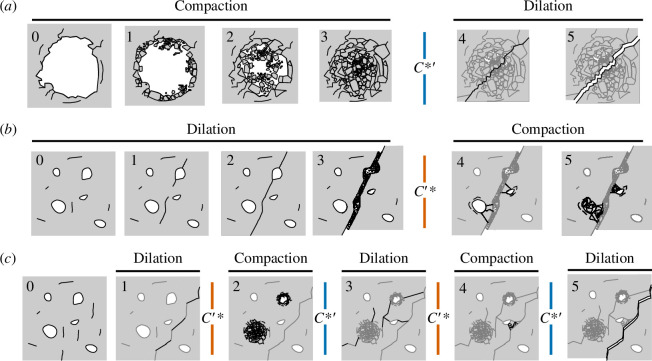
Micromechanical deformation mechanisms. (*a*) Deformation initially characterized by cataclastic pore collapse. During the ‘compaction’ sequence (0−3), a porous sample (porosity shown in white) is subject to loading. Stress concentration drives cataclasis and pore collapse. Beyond the C*’ threshold, the primary micromechanisms are the generation, growth and coalescence of fractures (4–5), driving sample dilation. (*b*) Deformation initially characterized by dilatant fracturing. In the ‘dilation’ sequence (0−3), slight porosity increase is driven by generation (0) and growth (1) of disconnected fractures. As these fractures link up (2, macroscopic failure), a through-running failure plane is created. Sliding on the fault plane leads to evolution of the fault core (3). In this scenario, the sample then undergoes the critical transition *C*’*, manifest in the progressive collapse of pores proximal to the fault zone (4–5). (*c*) Deformation initially characterized by dilation, before transitioning between dilation- (1, 3 and 5) and compaction-dominant (2, 4) mechanisms. In this scenario, the sample alternately meets C*ʹ and *C*ʹ*.

In figure 6, we summarize the operative micromechanical processes discussed in this section, as informed by our experimental and microstructural data. Figure 6*a* depicts a pore (0) subject to cataclastic pore collapse as a function of ongoing deformation (1–3). Bulk sample porosity decreases relative to the initial state as the porous network is progressively occluded. Beyond a threshold stress ( $C^{*}\prime$ ) fractures nucleate and grow (4–5), driving some increase in porosity (although not beyond the initial state). This scenario is reflective of sample R10−12, as shown in figure 3*a*. In figure 6*b*, dilatant processes are illustrated: first distributed microcracking (1), then growth and coalescence of these fractures into a localized failure plane (2), which can evolve to become a complex fracture zone (3). This progression is associated with a marked increase in porosity. Beyond the critical stress state $C\prime^{*}$ , cataclastic pore collapse is promoted in the vicinity of the fracture zone (4), which may be reflected by a decrease in sample porosity (5). This scenario is reflective of sample R10−15, as shown in figure 4*a*. Finally, in figure 6*c*, we illustrate the proposed process leading to deformation signals such as that shown in figure 5: repeated post-failure switching between compaction-dominant and dilation-dominant deformation mechanisms. In this case, porosity fluctuates around the initial value throughout deformation, as the primary mechanism alternates between fracture generation and propagation (1, 3 and 5) and cataclastic pore collapse (2 and 4).

## 4. Mechanical deformation and fluid flow

**Figure 7.**
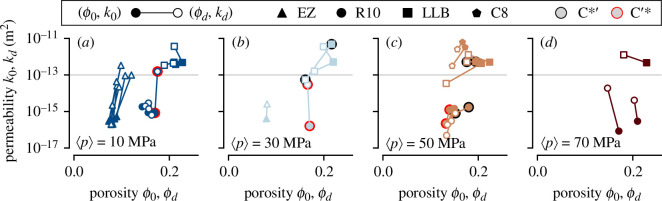
Pre- and post-deformation porosity and permeability shown in solid and open symbols, respectively, for new and compiled andesites. Samples which have undergone $C\prime^{*}$ or $C^{*}\prime$ are highlighted by light red or black circles, respectively. (*a*) Samples deformed at ⟨p⟩= 10 MPa . (*b*) Samples deformed at ⟨p⟩= 30 MPa . (*c*) Samples deformed at ⟨p⟩= 50 MPa . (*d*) Samples deformed at ⟨p⟩= 70 MPa . Colours correspond to effective pressure, as in previous figures.

Prior to deformation, we performed measurements of porosity ( $\phi_{0}$ ) and permeability ( $k_{0}$ ) on all samples. The post-deformation permeability $k_{1}$ was measured under identical conditions after unloading the samples. It is worth highlighting that this procedure allows comparison between samples with different deformation histories; however, the *in situ* permeabilities will probably be lower due to the differences in confining pressure for the permeability measurements (1 MPa) and the deformation experiments (as listed in table 1). Using the porosity change signal, the post-deformation porosity can be calculated ( $\phi_{1}=\phi_{0}+\delta \phi_{i}$ ). Figure 7 shows pre- and post-deformation porosity and permeability for the new data of this study, alongside andesites from Volcán de Colima from prior studies; these published examples are distinguished by their sampling localities, El Zarco (EZ), San Antonio (C8) and La Lumbre (LLB) and encompass a range of initial porosities and deformation behaviours. Note that LLB-13 is an andesite studied in [35], whereas LLB-2 is a sample prepared from the same block, new to this study. Plotted mechanical data are separated by effective pressure: 10 (figure 7*a*), 30 (figure 7*b*), 50 (figure 7*c*) and 70 MPa (figure 7*d*).

Results from previous data are summarized as follows. Low- $\phi_{0}$ , low- ⟨p⟩ deformation tends to result in large increases in permeability, and relatively little increase in porosity, e.g. EZ andesite (figure 7*a*). This is attributed to brittle failure establishing a flow path (i.e. a macroscopic fracture), which remains a highly efficient permeable pathway [54] irrespective of subsequent cataclastic mechanisms (in particular the generation of friction-induced fault gouge) [32]. High- $\phi_{0}$ , high- ⟨p⟩ deformation tends to result in decreases in porosity and permeability. For example, C8 andesite exhibits a concomitant decrease in both properties (figure 7*c*). Compaction in this sample is manifest as distributed cracking and pore collapse interspersed with localized planes or layers of collapsed pores (i.e. compaction bands) oriented sub-perpendicular to the direction of fluid flow [17]. Higher porosity andesites (LLB, $\phi_{0}\sim 0.22$ ) exhibit a reduction in porosity under all investigated conditions ([35]; figure 7*a–d*), but permeability change is inconsistent, exhibiting either an increase or decrease typically of less than one order of magnitude. Counter-intuitively, low amounts of compaction may increase permeability in volcanic rock as microcracks connect otherwise isolated porosity [35], a phenomenon that can be overprinted at high strain by global reduction in sample porosity. Moreover, compaction localization may not always constitute an effective barrier to fluid flow due to their tortuous and non-contiguous form [17].

At effective pressure $\left\langle p\right\rangle =$ 50 and 70 MPa, the R10 andesite samples of this study agree with previous data (cf. LLB andesite, figure 7*c,d*), and emphasize the fact that permeability may increase following compaction. As highlighted by Heap *et al*. [17], the tortuous and non-contiguous geometries of compaction bands—a function of the complex pre-existing pore geometries and variable crystallinity—drive only moderate porosity reduction (0.04−0.08 reduction within the compaction bands) compared with similar features in sandstones (approx. 0.15% [67]). This observation is in line with other studies identifying compaction bands in volcanic rocks [16,17,22,23]. Thus, at higher ⟨p⟩ , the effect of deformation is strongly dependent on both the angle and relative permeability of compaction localization (see also [67]). Microstructural evidence indicates that at intermediate sample porosities, compaction bands in volcanic rock can be oriented at oblique angles to the sample axis, rather than perpendicular as observed in other materials [67]. This is proposed to be dependent on sample heterogeneity [17]: pore size shape and distribution in these andesites is complex and variable, which strongly influences their physical and mechanical properties [44]. Moreover, the non-contiguous and diffuse nature of compaction bands in volcanic rock is in contrast to the discrete features sometimes observed in sedimentary rocks [68]. Interestingly, the compilation reveals that post-failure transition from compaction to dilation ( $C^{*}\prime$ ) does little by way of increasing sample permeability (see samples highlighted in black in figure 7*b,c*). This is presumably due to the large amounts of strain required to achieve this transition (figure 3*b*), which results in substantial comminution of groundmass (figure 3*c*). The macroscopic fracture zone imposed at $C^{*}\prime$ is generally not oriented favourably to fluid flow ( θ~π/2 ; figure 3*d*). Nevertheless, permeability reduction in samples having undergone $C^{*}\prime$ is not as pronounced as in samples that were unloaded before this critical stress state was reached, highlighting the contribution of dilatant shear fracture to the effective sample permeability.

At lower effective pressure ( $\left\langle p\right\rangle =10$ MPa, figure 7*a*), changes to the porosity and permeability of the R10 andesite samples tend to be subtle. We infer that the highly irregular pore shapes and high crystal cargo [38] impedes the formation of a geometrically simple fracture; rather, macroscopic failure is manifest as a tortuous network of interconnected pores. The exception is the sample that has achieved the critical stress state $C\prime^{*}$ (see figure 4): although there is only slight net dilation, permeability increases by approximately two orders of magnitude (highlighted in red on figure 7*a*). This is echoed in figure 7*b* for a sample deformed at $\left\langle p\right\rangle =30$ MPa (highlighted in red): in this case the sample porosity has decreased, but permeability has been enhanced by two orders of magnitude, again having surpassed $C\prime^{*}$ . Seemingly, this complex post-failure behaviour, which is (to an extent) self-limiting in terms of net porosity change (figure 5) is a mechanism for the generation of highly efficient fluid flow pathways. As shown in figure 4*c,d*, fluid flow paths may be generated which are oriented θ~π/4 (approx. 45°) relative to the sample axis and direction of fluid flow.

Much of the variability in the effect of deformation on permeability can be understood by considering the post-deformation samples as a simplified two-dimensional layered medium. A sample of dimensions $L\times \sum_{i}^{n}w_{i}$ , where L is length and $w_{i}$ corresponds to the width of each layer i , can be characterized by its arithmetic and harmonic mean permeabilities ${\langle k}_{x}\rangle =\left(\sum_{i=1}^{n}w_{i}k_{i}\right)/L$ and $\left\langle k_{z}\right\rangle =L/(\sum_{i=1}^{n}w_{i}/k_{i})$ , respectively [69]. Layer permeabilities are described by $k_{i}$ . Assuming the sample comprises a host medium of permeability $k_{0}$ , containing a planar feature (‘inclusion’) of permeability $k_{1}$ (which may be higher or lower than $k_{0}$ ), we can determine both the effect on equivalent permeability ⟨k⟩ of varying $k_{1}$ and the angle θ of the inclusion with respect to fluid flow via


(4.1) $$\left\langle k\right\rangle =\left\langle k_{z}\right\rangle sin^{2}\left(\theta \right)+\left\langle k_{x}\right\rangle cos^{2}\left(\theta \right).$$

**Figure 8.**
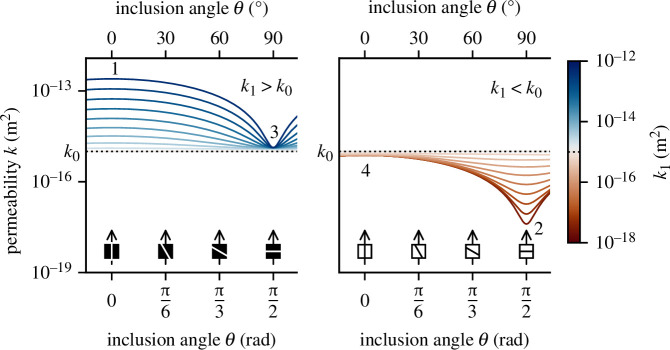
The effect of inclusion angle on equivalent permeability $\left\langle k\right\rangle$ . Following equation 4.1, $\left\langle k\right\rangle$ is calculated for a range of values of $k_{1}$ and inclusion angle with respect to the direction of fluid flow ( θ ), while all other variables are kept constant (host permeability 𝑘_0_ = 10–15 m^2^ (dotted line), sample length L=40 mm; inclusion layer width $w_{1}=5$ mm; host layer width $w_{0}=20–w_{1}$ mm). $\left\langle k\right\rangle$ is shown by the coloured lines, corresponding to $k_{1}$ . Panels are separated according to whether $k_{1}>k_{0}$ or vice versa. Points 1−4 are mentioned in the text.

We visualize equation 4.1 in figure 8, assuming a two-layer medium of dimensions $L\times (w_{0}+w_{1})$ . Values of 40, 15 and 5 mm are imposed for L , $w_{0}$ and $w_{1}$ , respectively, and we use a permeability value for the layer $w_{0}$ of $k_{0}=10^{-15}$ m^2^. Adopting a range of $k_{1}$ from $10^{-18}$ to $10^{-12}$ m^2^ for the layer (inclusion) $w_{1}$ allows us to calculate $k_{x}$ and $k_{z}$ , and in turn equivalent permeability $\left\langle k\right\rangle$ after equation 4.1. In figure 8, the values of $\left\langle k\right\rangle$ as a function of inclusion angle are shown by the coloured lines. From equation 4.1, it follows that if $k_{1}\gg k_{0}$ (i.e. considering a through-running macroscopic fracture), then equivalent permeability will be substantially augmented parallel to the orientation of the inclusion (low θ ; see point 1 on figure 8). At $k_{1}\ll k_{0}$ (e.g. considering a dense compaction band), permeability will be greatly diminished perpendicular to the orientation of the inclusion (point 2 on figure 8). At $k_{1}≳k_{0}$ or $k_{1}≲k_{0}$ , changes to equivalent permeability will be slight. Irrespective of the magnitude of $\left|k_{0}-k_{1}\right|$ , any increase or decrease in $\left\langle k\right\rangle$ is strongly dependent on the orientation of the inclusion [69]: a highly permeable fracture oriented perpendicularly to fluid flow ( θ=π/2 ) or a low-permeability compaction band oriented parallel to fluid flow ( θ=0 ) will not deviate $\left\langle k\right\rangle$ far from $k_{0}$ (i.e. 3 and 4 on figure 8). In our experiments, post-failure samples fall on a spectrum from $k_{1}\gg k_{0},\theta =0$ to $k_{1}\ll k_{0},\theta =\pi /2$ , primarily as a function of increasing effective pressure ⟨p⟩ . From our microstructural data (see also [17]), we posit that compaction localization features in these andesites can rarely be approximated as being oriented at π/2 with respect to the sample axis (rather, they may be closer to π/3 ). Interpreting figure 8 in this context yields the insight that absolute changes in permeability as a function of initial localized failure in porous volcanic rocks is likely to always be greater in the brittle field than the ductile field. Investigating the role of inter-sample heterogeneity, quantifying relevant flaw length scales and their spatial distributions, and in particular the upscaling of these observations beyond the centimetric scale to examine the effects of mechanical deformation on fluid flow are key avenues for future research. Deformation of synthetic or numerical samples [64,70,71], syn-deformation imaging [72–74] and the use of equivalent medium permeability models [69] each hold promise for shedding light on these complexities.

## 5. Implications of the mechanical limit

Our results point to functional limits to both compaction and dilation in natural geological systems. Moreover, we reveal the importance of considering post-failure deformation behaviour, which can significantly modify fluid flow. The samples in this study for which we demonstrate the most complex post-failure deformation contained an initial porosity of approximately 0.16. Compilations of edifice-forming pyroclasts [10,42,43,75] often reveal modal porosities in the range 0.1<ϕ<0.2 ; in line with estimates of edifice porosity from gravimetric inversion [76]. Thus, the results presented here are highly relevant to materials that comprise a volumetrically important proportion of a volcanic edifice. Moreover, samples were deformed herein at effective pressures ranging from 10 to 70 MPa. Lithostatic pressure at depth $p\left(z\right)$ is a function of atmospheric (datum) pressure $p_{0}$ , surface gravitational acceleration g and bulk rock density $\rho \left(z\right)$ ; the relation $p\left(z\right)=p_{0}+g\int_{0}^{z}\rho \left(z\right)dz$ indicates that the imposed values of $\left\langle p\right\rangle$ represent depths from approximately 400 to 3000 m ( ρ = 2320 kg m^−3^), relevant to shallow crustal processes. This is especially pertinent considering the high stresses and strain associated with magma migration-induced uplift, the ascent and extrusion of lava and related deformation processes.

**Figure 9.**
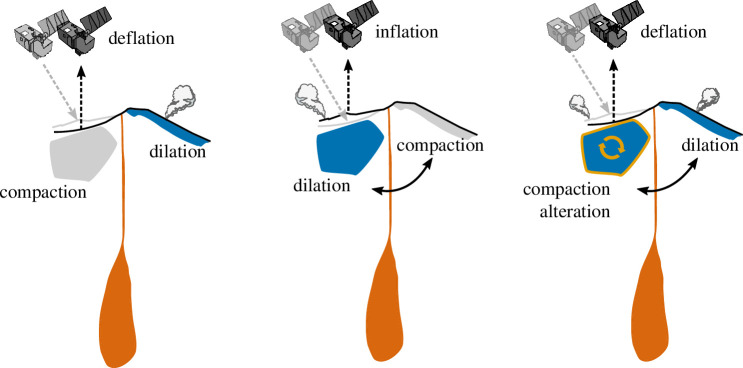
Illustration of the effects of failure mode transitions on volcanic outgassing and observed surface deformation. In the first instance (left panel), a zone of the shallow volcanic edifice is undergoing dilatant deformation, allowing fumarolic outgassing from one flank. A zone of subsurface compaction is manifest as edifice deflation as inferred from ground displacement measurements. Switches in failure mode (centre panel) lead to a reversal in the observed deformation signal and activation of a different fumarole area. In the right panel, a further compaction–dilation/dilation–compaction switch means that the displacement observations revert to their initial state. In this scenario, outgassing is now distributed across both flanks, as permeable fracture networks have been developed and activated in both locations. Circulation of hydrothermal fluids has been promoted, allowing alteration to occur.

Deformation of volcanic edifices, including non-magmagenic deformation, is common [77]. Indeed, in a study that systematically monitored 198 volcanoes over 3 years [78], there were more ‘false positives’ than ‘true positives’, which is to say that the incidence of volcanoes remaining quiescent following measurable deformation was higher than volcanoes which erupted after deformation, attributed in part to complexity arising from local stress fields. At many volcanoes, ground deformation changes—detected using global navigation satellite systems (GNSS) or interferometric synthetic aperture radar (InSAR), for example—exhibit variations in rate and/or direction over relatively short timescales (e.g. days or years) [79–81]. A mechanical limit for dilatant and compactive processes in a volcanic edifice, as we describe herein, provides an explanation for alternating surface deformation observed in GNSS or InSAR data that may be seemingly uncorrelated with magmatic activity (figure 9).

Inversion modelling, which typically requires surface displacement data as an input, has indicated that the strength and volumetric response of edifice rock to loading influences the dynamics of magmatic processes. For example, edifice-scale modelling of displacement at Piton de la Fournaise (La Réunion) necessitated a maximum stress accumulation value in the upper edifice, with the authors proposing that nonlinear behaviour and stress periodicity are promoted by the existence of this critical threshold [82]. In turn, this explains complex distal magma migration and allows the volcanic edifice to modulate magma transfer from depth—a so-called ‘valve effect’ controlled by the mechanical evolution of the edifice [82]. Our experimental data support such a mechanism at volcanoes more generally, such that micro- and meso-scale mechanical limits to volumetric change are echoed at the scale of the volcanic superstructure (cf. kinematic volcano-tectonic coupling [83] or co- and post-seismic reversals in compressional and extensional quadrants at the field scale [84,85]). Notably, a further study was able to effectively model surface displacements at Piton de la Fournaise by accounting for an ‘incremental damage’ factor [86], underlining the importance of accounting for ongoing edifice deformation when modelling magma migration based on surface displacement data.

Comparable self-regulating mechanisms have also been proposed to govern near-surface gas loss in volcanic systems [87,88], whereby the time-dependent outgassing capacity of tuffisite networks is reflected in localized fumarole activity. In a similar vein, we suggest that repeated transitions in deformation style throughout the edifice could lead to transient activation and deactivation of discrete outgassing fields (figure 9). A corollary of this is that hydrothermal circulation in the subsurface may be periodically enhanced, promoting alteration [89], mass transfer [1,3] and epithermal ore formation [90] (figure 9). Alteration can in turn be associated with volcanic hazards [91–93]. Volcano-drilling projects have shown that the upper conduit and shallow edifice of silicic to intermediate volcanoes is often intensely damaged. For example, drill cores of Unzendake [29] and Nigorikawa Caldera [30] preserve polymict volcanic breccia rather than intact pristine lava, suggestive of a complex stress–strain history and potentially indicative of multiple overprinting of brittle and ductile deformation mechanisms. The low permeability of exhumed Unzendake drill cores (of order 10^−19^−10^−17^ m^2^ [94]) belies the fact that they must have allowed abundant hydrothermal circulation in their history [95,96], resulting in extensive alteration [97]. Our experimental results emphasize the complex coupling between mechanical deformation and fluid flow in the shallow crust.

## 6. Conclusions

When subject to applied differential stress, rocks can fail in a dilatant (brittle) or compactant (ductile) manner. However, when deformation continues beyond the initial failure threshold—as may occur in natural crustal settings—the dominant deformation behaviour (dilatant or compactant) may switch. In order to investigate the effect of complex post-failure deformation on the physical rock properties, we conduct a series of triaxial deformation experiments on samples of porous andesite from Ruapehu volcano, Aotearoa New Zealand. This material was selected because its porosity range (approx. 0.14−0.20) is typical of modal porosities of edifice-forming rocks, and it straddles the transition between positive and negative volumetric responses to compression when deformed under effective pressures relevant to the shallow crust (i.e. the inelastic compaction factor $\delta \phi_{i}\epsilon_{i}^{-1}$ tends towards zero under differential stress).

As well as demonstrating a post-failure switch from compactant to dilatant behaviour (a phenomenon known as $C^{*}\prime$ ), we describe for the first time a critical stress state whereat a switch from dilatant to compactant behaviour is observed. We refer to this as $C\prime^{*}$ , and demonstrate its occurrence both through new deformation experiments and reappraisal of published experimental data. Critically, samples that achieved this stress state, under volcanically relevant deformation and pressure conditions, exhibited substantial increases in permeability. This is in contrast to samples that undergo $C^{*}\prime$ , where any dilatancy-induced enhancement in permeability is effectively masked by the global decrease in sample porosity—a consequence of the high strains generally associated with achieving this transition. Additionally, we show that repeated transitions between dilatant and compactant behaviour are possible. We infer that this reflects a competition between compactant mechanisms (e.g. distributed cracking, cataclastic pore collapse) and dilatant mechanisms (e.g. fracture generation, coalescence and fault sliding), with the dominant process being governed by localized stress changes. We infer that these behaviours are controlled by effective pressure, porosity and sample microstructural heterogeneity.

We emphasize that complex post-failure deformation processes could have important ramifications for the edifice-scale deformation and evolution of gas pressure at shallow depths in volcanic systems—a critical determinant of explosive volcanic activity—as well as influencing thermal and solute transport in the shallow crust. In addition to influencing volcanic hazard, these parameters govern the distribution and abundance of commercially and industrially relevant minerals and metals in porphyry ore deposits, the long-term viability of carbon capture and storage reservoirs and nuclear waste storage in the crust, and the stimulation and productivity of geothermal reservoirs—all of which are key to tackling societal problems in the foreseeable future.

## Data Availability

All new data and the Python code used for analysis and plotting are provided in the GitHub repository https://github.com/jifarquharson/failure-mode-switching, archived via Zenodo at https://doi.org/10.5281/zenodo.1323692 [98]. Supplementary material is available online [99].
